# The Principles and Practice of Obstetric Medicine and Surgery, in Reference to the Process of Parturition

**Published:** 1842-01-29

**Authors:** 


### BIBLIOGRAPHICAL NOTICES.

*The Principles and Practice of Obstetric Medicine and Surgery, in reference to the Process of Parturition. Illustrated by one hundred and forty-two figures.* By Francis H. Ramsbotham, M. D., &c. &c. First American Edition, Revised. Philadelphia. Lea & Blanchard. 1842. pp. 458. Royal Octavo.

This work is one of the most useful lately issued from the American press. It is a reprint of Rambotham's work, with his excellent illustrations. These comprise a series of fifty-two plates, containing one hundred and forty-two figures. They are beautiful lithographs, executed in admirable style, under the careful supervision of the publishers, and are amply sufficient for all purposes of the obstetric student and practitioner. The text and description of the plates is written in a clear and simple style, and is extended enough for the purpose.

In common with many members of the profession, we have long regretted the absence of a work of this kind. The mechanism of labour is simplicity itself, when illustrated with plates and models; but one of the most unintelligible departments of the art of medicine, when taught only in words, or studied from works of midwifery. There are two distinct divisions of the subject; that connected with labour as a physiological and mechanical action, and the treatment of the disorders incident to it. The object of the present work is to explain the process of parturition, which it has done more satisfactorily than any other of equally moderate dimensions and cost.

